# Chikungunya-related Fatality Rates, Mauritius, India, and Reunion Island

**DOI:** 10.3201/eid1408.080201

**Published:** 2008-08

**Authors:** Philippe Renault, Loic Josseran, Vincent Pierre

**Affiliations:** * Cellule Interrégionale d'Épidémiologie Réunion-Mayotte, Saint-Denis, Réunion, France; † Institut de Veille Sanitaire, Saint-Maurice, France

**Keywords:** chikungunya, fatality rate, Indian Ocean, epidemic, letter

**To the Editor:** During the epidemic of chikungunya virus infection that occurred on Reunion Island in 2005–06, we reported an overmortality corresponding to the epidemic peak, which was estimated by comparing observed and expected deaths (*1*). The excess was similar to the number of deaths related to chikungunya infection reported by death certificates (*2*). The case-fatality rate (CFR) on Reunion Island was estimated to be 1/1,000 population.

According to Beesoon et al. (*3*), the fatality rate attributable to chikungunya infection was much higher on Mauritius: 743 deaths in excess of expected deaths led to a CFR of ≈4.5%, with 15,760 confirmed or suspected cases for 2005 and 2006 as reported in this letter. A similar CFR of 4.9% can be calculated for the city of Ahmedabad, India, during the 2006 chikungunya epidemic (*4*).

This 45- to 49-fold difference could be explained by a greater severity of chikungunya infection in Mauritius or Ahmedabad that could be due to a mutating strain, differences in the preexisting conditions of patients, differences in the management of patients, or by coincident deaths in excess from other causes.

However, the most probable explanation can be attributed to the surveillance systems of chikungunya cases. On Reunion Island, surveillance was highly sensitive and relied either on active case finding or on estimates of suspected cases. Results have been assessed by iterative external studies and serosurveys, and the CFR we found is likely consistent.

If we apply this rate to Mauritius, ≈60% of the population would have contracted chikungunya infection during this epidemic. If so, the risk of epidemic resurgence could be much lower than previously expected. This point raises the need to conduct seroprevalence studies in those territories, the only way to evaluate the herd immunity level of the population.
